# The “new normal”: Adapting doctoral trainee career preparation for broad career paths in science

**DOI:** 10.1371/journal.pone.0177035

**Published:** 2017-05-24

**Authors:** Rebekah St. Clair, Tamara Hutto, Cora MacBeth, Wendy Newstetter, Nael A. McCarty, Julia Melkers

**Affiliations:** 1 School of Public Policy, Georgia Institute of Technology, Atlanta, GA, United States of America; 2 Atlanta BEST Program, Emory University and Georgia Institute of Technology, Atlanta, GA, United States of America; 3 Emory College of Arts and Sciences, Emory University, Atlanta, GA, United States of America; 4 College of Engineering, Georgia Institute of Technology, Atlanta, GA, United States of America; 5 School of Medicine, Emory University, Atlanta, GA, United States of America; Waseda University, JAPAN

## Abstract

Doctoral recipients in the biomedical sciences and STEM fields are showing increased interest in career opportunities beyond academic positions. While recent research has addressed the interests and preferences of doctoral trainees for non-academic careers, the strategies and resources that trainees use to prepare for a broad job market (non-academic) are poorly understood. The recent adaptation of the Social Cognitive Career Theory to explicitly highlight the interplay of contextual support mechanisms, individual career search efficacy, and self-adaptation of job search processes underscores the value of attention to this explicit career phase. Our research addresses the factors that affect the career search confidence and job search strategies of doctoral trainees with non-academic career interests and is based on nearly 900 respondents from an NIH-funded survey of doctoral students and postdoctoral fellows in the biomedical sciences at two U.S. universities. Using structural equation modeling, we find that trainees pursuing non-academic careers, and/or with low perceived program support for career goals, have lower career development and search process efficacy (CDSE), and receive different levels of support from their advisors/supervisors. We also find evidence of trainee adaptation driven by their career search efficacy, and not by career interests.

## Introduction

There is a growing trend among doctoral trainees in the sciences to pursue careers outside of academia [1, 2, 3, 4]. Changes in the size of the PhD workforce and corresponding changes in the job market present a very different career landscape than in prior decades, with evidence of an increasing interest in careers beyond the professoriate [5]. A recent National Science Foundation (NSF) report on workforce trends among doctoral recipients additionally shows continued decline in the academic employment rates in life sciences, physical sciences, and engineering, with a rising number of PhD recipients in the life sciences (including biomedicine) holding jobs in industry (31%) ([3], p.8). Whether driven by the job market or by personal preferences, there is an observable shift in the career outcomes of PhD scientists [1, 6, 7]. A growing body of research has focused on explaining these interests, shedding light on the drivers of what seems to be a change in the acceptable and desirable pathways for PhD scientists [4, 8, 9]. Yet, a gap exists in our understanding of how doctoral trainees interested in non-academic careers adapt their career search strategies for non-traditional pathways, and the role that institutions play in this adaptation.

At issue is the concern expressed by doctoral students that they are trained only for careers in academia, suggesting a lack of preparedness for careers outside of the academic marketplace [2, 10]. This is exacerbated by the fact that career development resources provided by universities are mostly intended to prepare doctoral trainees specifically for academic careers [6, 11, 12]. This practice often encourages or reinforces existing mentoring practices that are geared toward academic pathways [13], which ultimately can leave doctoral recipients with non-academic career interests left on their own to locate the resources that they need to succeed in these broader job markets.

The purpose of our research was to assess the career preparation strategies of biomedical doctoral trainees with non-academic career interests. With the recently adapted Social Cognitive Career Theory model, together with Social Capital Theory, as our foundation, we focused explicitly on career search and development. Using structural equation modeling (SEM), we examined the role of “process efficacy” of these trainees as a mediating factor in their career development activities. Process efficacy is “the perceived ability to manage specific tasks necessary for career preparation, entry, adjustment, or change across diverse occupational paths” [14]. As suggested by Lent et al. [15], self-efficacy related to job search behaviors has been found to be a mediating factor in these actions. The SEM method allowed us to understand whether differences in career development exist depending on one’s career goals and efficacy. Because career development typically involves the support of others, we also explored within our SEM models how doctoral advisors and other institutional support systems matter in this process. Data for our study are drawn from an NIH-funded survey of doctoral students and postdoctoral fellows in biomedical disciplines at two U.S. academic institutions.

### The social cognitive career model

Studies of post-secondary trainee career interests often draw upon Lent, Brown, and Hackett’s [16] *Social Cognitive Career Theory* (SCCT) which holds that an individual’s career choice stems from their background (demographics, family education, etc.), as well as from their learning experiences, which in turn drive their self-efficacy and career outcome expectations [14, 17]. A recent adaptation of the SCCT model [14, 15] presents a model of process and adaptive behaviors known as the career self-management model. Here, the assumption is that individuals engage in certain behaviors “to help direct their own career (and educational) development, both under ordinary circumstances and when beset by stressful conditions”, implying both reactive and proactive career development behaviors ([14], p. 559). This adaptation of the SCCT has been recently applied to a distinctive transition point in career development: the job search process. Here, general self-efficacy (a type of self-confidence) in the general model of career management has been more finely specified as “job search self-efficacy”, meaning a “source” of outcome expectations that influences job search behaviors, through mediating other factors like support [18]. With few exceptions (e.g. [19, 20]), the literature has focused mostly on career outcomes and has largely neglected to study the process components of the SCCT [14]. For studies of non-academic career aspirations in science, this means that *we do not yet understand the strategies and resources that trainees use to prepare for the non-academic job market*, *nor do we understand what determines the ability of trainees to navigate the process*.

**Fig 1:**
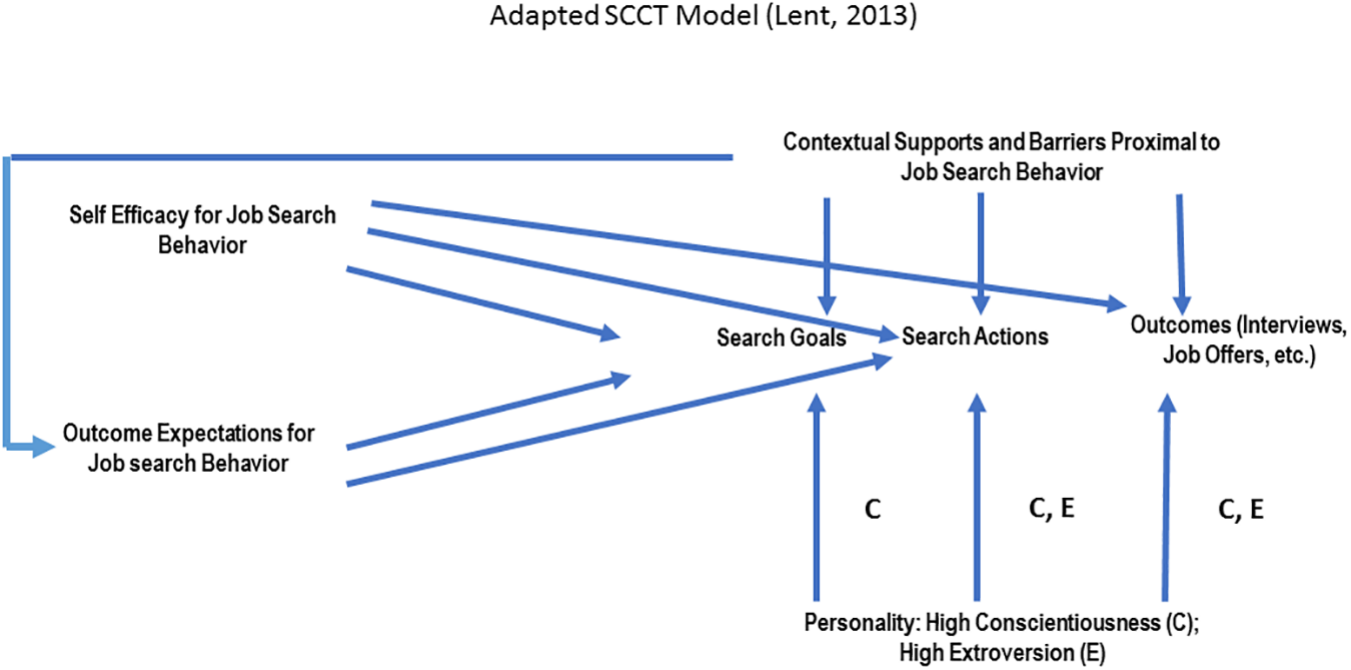
Adapted SCCT model.

Given these research gaps in understanding the process of career development for doctoral trainees, we seek to understand the factors that drive trainees to adapt and assume responsibility for their non-academic career aspirations, or as Lent and Brown [14] described it–understand the factors “that lead people to enact behaviors that aid their own educational and occupational progress”. Specifically, we ask: *what factors (if any) are driving doctoral trainees to adapt their career development processes to seek out resources*, *including but not limited to*, *their advisor*? Further, provided that theory has predicted [14] and demonstrated [21] that career development behaviors are determined by perceptions of self- and/or career efficacy, we also ask: *is career search efficacy a mediating factor in the decision of a doctoral trainee to seek advice from their advisor*, *as well as pursue different career development strategies*? The theoretical logic of this analysis builds on the newly expanded SCCT and is presented in Fig 1.

In the doctoral setting, institutional context includes important career development social capital resources, such as one’s advisor and other faculty as well as peers. Social Capital Theory is based on the notion that capital (resources) is gained through social relations, hierarchical structures, and other factors [22, 23]. In doctoral programs, advisors as well as other faculty and professionals can be social capital resources (or barriers) for trainees pursuing non-academic career outcomes. Given this, we conceptualize the context in the SCCT Model as career-relevant social capital, which conveys the role of the academic environment in which the trainee functions. By understanding the social capital of doctoral trainee career development, we can better understand the contextual supports and barriers to STEM doctoral career search efficacy.

### Institutional context, culture, and trainee career search efficacy

Academic institutions provide the contextual support for career development through career service offices and other support systems, but these resources often are not tailored for doctoral students [13]. This omission may serve as a barrier of sorts [24]. For doctoral trainees, faculty advisors have traditionally played a central and instrumental role in their career development and training [10, 25, 26, 27]. Shaped by embedded academic cultures, doctoral advisors tend to prepare their trainees for academic career paths. This suggests both a lack of necessary skills for trainees seeking a career beyond the professoriate, as well as a lack of resources for trainees to develop these skills [6, 13, 24, 28, 29, 30].

Notably, lack of support for non-academic careers may be less direct, or even unintentional, as faculty are often described as most comfortable in “cloning” themselves [31], affecting the type of advice that they provide. Most faculty have limited experience in careers outside of academia [32] and also may feel ill-equipped to provide sound advice on non-academic careers. Pressures also exist for faculty, where their own success is often judged in part on the placement and academic productivity of their trainees, again shaping their approach to training [33]. Evidence of passive training, where trainees model their own work on that of their advisor [34, 35], also transmits the norms of academic culture into the mentoring and support of trainees, leaving trainees with non-academic career interests with nothing to model. A possible exception is in the case of faculty with strong ties to industry and in cases where doctoral research is supported by industry, increasing the likelihood of non-academic career placement [6, 7]. However, these advisor industry-social capital resources (i.e. professional industry connections) may not be equally accessible to trainees [23].

How trainees perceive their advisor and other career development resources motivates their job search behaviors [21]. Mangematin [6] argues that the relationship between an advisor and an advisee is synonymous to a contract, where the career interests and activities of trainees are expected to align with the advisor’s expectations in exchange for training, resources, and career development assistance. When a “psychological contract” has been breached, individuals have been found to often turn to more external sources of career self-management [20]. In other words, the traditional preparation of doctoral trainees may not always be (or perceived to be) as effective for non-academic career preparation, compelling doctoral trainees to seek alternative resources to help them to explore and prepare for non-academic career paths.

The newly adapted SCCT highlights an important facet of career development–that individuals may have to adapt their career development strategies based on what support and contextual challenges exist, and that this adaptation will increase their career capacity. This model emphasizes the unpredictability of individual work lives, where individuals face less familiar or “unusual career challenges” [14] potentially requiring them to take different strategies to achieve their career goals. If local resources (advisors and institutional) are not in support of a trainee’s career interests, trainees may face uncertainty on how to best prepare for, explore, and find jobs outside of the academic marketplace. Lent and colleagues [14, 15] call this self-management where the individual takes control of their career direction, including in the job search and other career preparation processes. Uncertainty can be a considerable weakness for doctoral trainees, signaling a lack of knowledge and confidence in how to prepare and acquire a career outside academia. Given this framework, *we expect that doctoral trainees with broad career interests that go beyond traditional academic jobs will have lower levels of confidence or self-efficacy in their career development and search process*, *and be less likely to seek out support from their primary advisor*.

### Self-management and adaptation in the career search process

If doctoral trainees with non-academic career interests must rely more on the self-management of their career development due to actual or perceived lack of support from the traditional sources of their advisors or institutions, they may adapt by seeking out other career development resources. In the SCCT model [14] these adaptive behaviors constitute career competencies that enable an individual to “cope with contextual challenges.” Adaptive behaviors increase self- and career efficacy [36], allowing individuals to gain more control over their career direction [21]. For doctoral trainees, this adaptive behavior may take many forms. Trainees may seek out explicit resources, materials or career training to prepare them for a non-academic career. They may also seek out individuals (i.e., peers or other non-academic professionals) for social capital resources such as advice and other support to help them enter the non-academic job market. Well established in the sociology literature, personal relationship and related social capital in job searching processes matters [37, 38, 39, 40], for both tangible and psychosocial outcomes.

Finally, as doctoral trainees self-manage their search and acquisition of career resources relevant to non-academic career interests, they may use different strategies depending on their career search efficacy (SCCT), as well as other factors relevant to how they process information [41]. What is unclear and poorly understood is how career preferences shape the types of resources that trainees pursue. For example, depending on one’s relationship, doctoral students and postdocs may attempt to reframe the discussion and seek the support from their established career-based ties (their advisor). For others, local (institutional or advisor) resources may not be available, alongside other means of resistance to trainees pursuing non-academic career outcomes. In SCCT terminology, this may be an attempt to adapt a known source and process to the non-career process. A striking and pro-active example of this self-management (and response to perceived inadequate career resources) is in the establishment of a doctoral student- and postdoc-led initiative to develop the Biotechnology and Life Science Advising (BALSA) Group which links doctoral recipients with biotechnology firms in the St. Louis, Missouri area [13].

Based on the established role of advisors and academic institutions in providing career development support, and the popularity of non-research/non-academic careers, we posit the following hypotheses:

*H1: the ability of doctoral students and postdocs interested in non-academic careers to adapt their own career search process to their own interests and needs will depend on their own career development and search self-efficacy (referred to here as CDSE)*.
*H2: the support received from their environment to pursue their career goals will be a driving factor in how students and postdocs adapt their career development strategies*.

## Materials and methods

The data for our research come from an on-going study of two Atlanta institutions (Georgia Institute of Technology and Emory University) that together comprise one of the 17 sites supported by the National Institutes of Health (NIH)-funded BEST (Broadening Experience in Scientific Training) Program. Atlanta BEST is designed to specifically prepare doctoral students and postdocs for a broad set of careers outside of the traditional academic pathway. This research has been approved by Georgia Tech’s Institutional Review Board (IRB#H13506), with reciprocal agreement by Emory University. Survey respondents provided informed consent and were then able to enter the survey.

### Data

The specific data used in this analysis came from two sources. First, the majority of the data come from a 2015 survey of doctoral trainees at the two Atlanta BEST institutions: Emory University and the Georgia Institute of Technology (Georgia Tech). The purpose of this survey was to understand the baseline interests and career search experiences of doctoral trainees in the biomedical sciences, allowing for a long-term comparison group of trainees affiliated with the Atlanta BEST program. For the purposes of our research, the survey provides a comprehensive set of survey data across several disciplines which allow us to address career search efficacy issues for trainees with both academic and non-academic career interests. All PhD students enrolled in a biomedical training program and postdoctoral fellows (BEST and non-BEST) on both university campuses in several biomedical disciplines were surveyed. Including postdoctoral fellows in our study was particularly important, as they remain a largely understudied population in the biomedical sciences [42].

This survey instrument was cooperatively designed across the 17 NIH-funded BEST programs and implemented online using Qualtrics software by Windrose Vision, a contract research and consulting organization. Lists of enrolled students and employed postdocs were obtained directly from the institutions and verified by central administration. Trainees received the survey and a series of reminders over several weeks. Response rates were strong: 1,705 graduate students and 667 postdocs in the two institutions were invited to the survey (including all Atlanta BEST trainees), and 892 responded, for a response rate of 38% (50% for postdocs and 33% for PhD students) across a variety of biomedical-related fields at Emory University and the Georgia Tech. The second source and remaining data for our study came from administrative data detailing participation in Atlanta BEST career development activities.

### Variables

The variables used in our analysis and the survey questions from which they derive are provided in Table 1. Critical to this analysis was whether trainees were interested in a traditional academic career, or have broader career interests. We included a variable to represent those individuals who were not interested in these broader (non-research and non-academic) careers. This was calculated by creating a dummy variable that distinguished those individuals who were “definitely pursuing” or “strongly considering” a Principal Investigator (PI) position in an academic institution *and* a research and teaching position at an academic institution (Cronbach alpha: 0.66), and those who were not.

**Table 1:** Variable and coding descriptions.

**Construct**	**Survey Questions and Related Coding**
**Career Interests**	
**Non-Research/** **Non-Academic Career**	Is the individual not strongly interested or definitely not pursuing a Principal Investigator (PI) position in an academic institution and a research and teaching position in an academic institution? (alpha 0.66)
**Institutional support**	
Perceived High Advisor Support	Does the individual perceive his/her advisor’s support for his/her career goals to be strong? Coded 1 if the individual “agreed” or “strongly agreed” with at least one of two statements regarding their advisor’s support. (alpha 0.84)
Perceived High Program Support	Does the individual perceive his/her department’s support for his/her career goals to be strong? Coded 1 if the individual “agreed” or “strongly agreed” with at least one of two statements regarding their department’s support. (alpha 0.79)
Career Search Efficacy	
**Confidence Total**	How strong is the individual’s career search efficacy? Summative variable of four career search skills (assessing skills and abilities to pursue desired career path, determining steps to pursue desired career path, identifying potential employers relevant to that career path, and achieving career goals) (alpha 0.84)
**Career Development Activities/Strategies**	
**Traditional Career Development Activities**	What career development activities have been pursued in the last year? 1) sought career advice from PI/advisor; 2) sought career advice from a faculty member other than advisor.
**Non-traditional Career Development Activities**	What career development activities (activities other than pursuing advice from advisor, supervisor, or other academic faculty member) have been pursued in the last year? Eleven different variables were assessed individually as non-traditional career development and adaptive strategies to improve career development: 1) held an internship within your institution, 2) held an internship outside your institution, 3) participated in job shadowing, 4) discussed career plans with professionals outside of academia, 5) read books, articles, and/or online sources about career development or planning, 6) discussed career plans with family, 7) attended a course about career planning for credit, 8) attended a course about career planning not for credit, 9) attended a career-related event at your institution (e.g., workshop, panel, career fair, seminar, etc.), 10) attended a career-related event not at your institution (e.g., workshop, panel, career fair, seminar, etc.) 11) participated in BEST activities.
Demographics and Background	
**Gender**	Is the individual a female?
Citizen	Is the individual a citizen?
**Minority Race**	Is the individual an underrepresented minority race/ethnicity?
**PhD Student**	Is the individual a PhD student or Postdoc?
**Institution**	What institution does the individual attend?
**BEST Trainee**	Is the individual a BEST trainee?

We identified two sets of dependent variables. First, Career Search Self Efficacy is central to the SCCT model, and to our research questions in this paper. To capture this, we created a summative variable composed of four career development variables on a five-point Likert scale about confidence in various career development activities as described further in Table 1 (Cronbach alpha: 0.84). This summative variable (“career search efficacy”) on a scale of 1–20 thus reflected the extent to which an individual was confident in their career search abilities.

Second, to fully understand the career development behaviors and actions of individuals at these institutions, we assessed a variety of career search strategies pursued. These included traditional sources of support, such as asking academic advisors and faculty members for advice, as well as less traditional, more adaptive career development behaviors, like seeking advice from professionals outside of academia. Additionally, and consistent with the adapted SCCT model, we included a variety of different categories of independent variables in our analyses. Specifically, eleven different career resource/strategy dependent variables were analyzed individually as non-traditional career development and adaptive strategies to improve career development, as shown in Table 1. Attempts to identify groupings of these items using Factor Analysis was not successful, suggesting independent types of career resource strategies. Thus, models were run for each individual career development strategy.

Perceived High Institutional Support was operationalized in two different ways: perceived overall program support for career goals and advisor support for career goals. Perceived High Program Support was a summative variable comprised of responses of “Agree” or “Strongly Agree” for two variables on a Likert scale, one asking about perceived strength of program support for career goals and the second asking about perceived strength of program support for steps taken to achieve those career goals (Cronbach alpha: 0.79). Similarly, Perceived High Advisor Support was a summative variable where respondents “Agreed” or “Strongly Agreed” that their advisor is supportive of their career goals and for the steps taken to achieve those career goals (Cronbach alpha: 0.82).

Our demographic and background variables include the individual’s race, gender, and citizenship, as well as their institution (Emory University or Georgia Tech) and whether or not they have participated in Atlanta BEST. This was important because the decision to participate in Atlanta BEST represents a career development/search strategy. Atlanta BEST has been well advertised and has a high level of visibility on both campuses. Participation in Atlanta BEST occurs at two levels. At the most intensive level, the Atlanta BEST program uses a cohort model, where trainees apply and are accepted into a two year program, and subsequently participate in a range of activities as a formal cohort. In addition to this formal affiliation, many Atlanta BEST activities are open to others on campus. Therefore, a second less intensive trainee affiliation exists where trainees select which events they would like to attend, and participate sporadically. We consider both levels of affiliation as BEST participation for this paper. Basic demographic variations in our data are provided in Table 2.

**Table 2:** Survey respondents.

**Trainee Survey Respondent Demographics** **(Percent Respondents)** **(n = 892)**	
**Female**	46%
**Male**	54%
**Underrepresented Minority**	13%
**U.S. Citizen**	62%
**PhD Student**	63%
**Postdoctoral Fellow**	37%
**Emory University**	59%
**Georgia Institute of Technology**	41%
**Atlanta BEST Program Trainees**	5%
**PhD Students**	4%
**Postdoctoral Fellows**	1%
**Other Atlanta BEST Participants**	17%

### Models and analysis

To address the adaptive career resource strategies of doctoral trainees, we constructed a structural equation model (SEM) [43] that allows us to understand the effects of career efficacy on trainee career search strategies, and whether these strategies differ by career goals (academic or non-academic). This method differs from traditional regression in a few keys ways. A main strength of SEM is handles complex relationships. Whereas in regression a researcher would have to conduct multiple and/or *ad hoc* analyses for additional effects, SEM allows us to disentangle the direct and indirect effects of academic versus non-academic career interests in one analysis [44]. For example, parental income may have a direct effect on adolescent academic achievement. However, it could also be that the effect of parental income on academic achievement may be mediated by home resources (i.e. a computer) that in turn affect academic achievement. In this case, parental income could indirectly affect academic achievement. In regression this would require multiple and independent analysis, whereas SEM incorporates all in a single model. Additionally, unlike in regression, SEM analyzes both observable and latent variables, which enables measurement errors of observed variables to be a part of the model and for factor analysis to be conducted alongside the testing of hypotheses. This allows for a more realistic and rigorous analysis [44].

**Fig 2:**
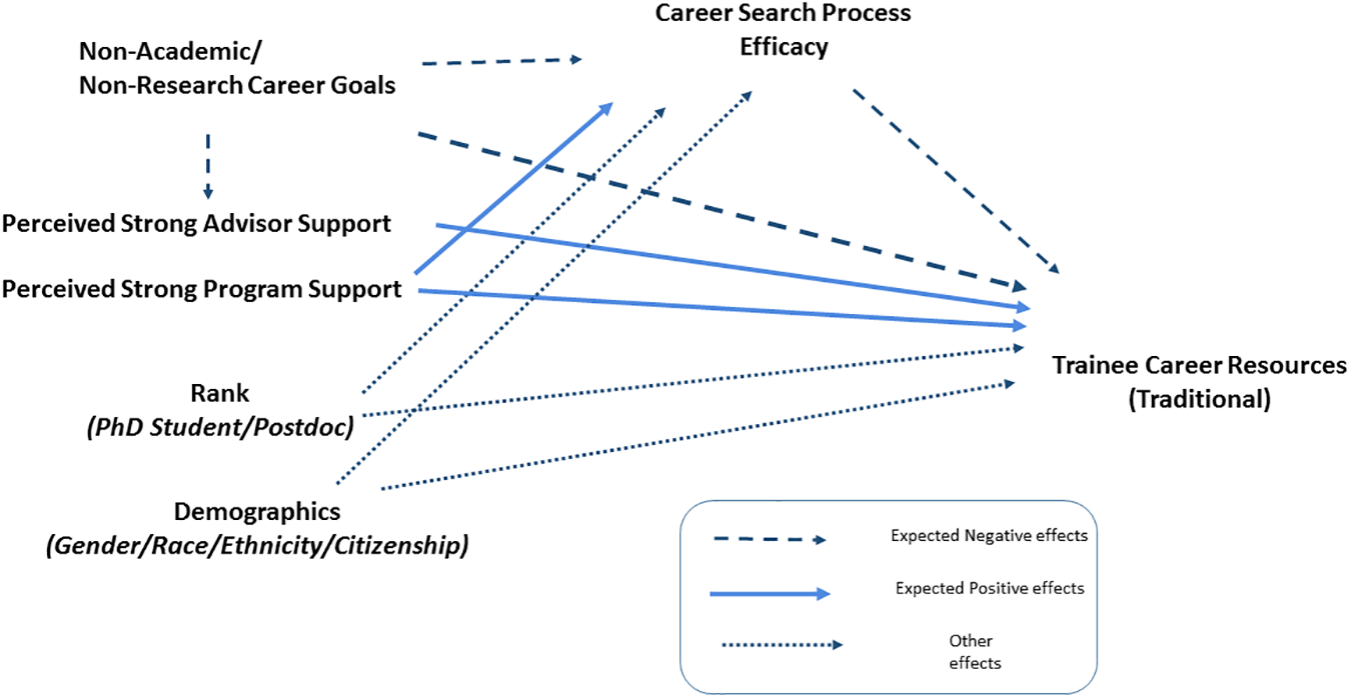
Structural equation model.

We used the “traditional” measure of model of fit–the chi-square statistic as our indicator. Each of our models failed to achieve a significant chi-square result at a 0.05 threshold, indicating our model has sufficient fit [45]. Given that research has shown how self-efficacy influences career choices and actions, this modeling approach enabled us to understand if and how self-efficacy mediates the relationship between individual and institutional characteristics on career development strategies. Importantly, it allows us to measure the direct impact of non-academic career interests on our key dependent variable, as well as the indirect impact it has on career search efficacy (Structural Equation Model shown in Fig 2). Overall, this analysis is useful both formatively (to Atlanta BEST) but also theoretically (newly adapted Social and Cognitive Career Theory).

## Results

### Descriptive results

Of the 892 respondents to the survey of trainees (Table 3), 59% are trainees at Emory University (41% at Georgia Institute of Technology), 63% are PhD students (37% are Postdoctoral Fellows), 46% are female, 62% are US citizens (born or naturalized), and 13% are members of an underrepresented minority group (African American, Hispanic, and/or Native Alaskan/American). Most striking, the vast majority of respondents (72%) are “definitely pursuing” or “strongly considering” non-academic/non-research careers. We find little variation across interest in academic versus non-academic careers by race/ethnicity.

**Table 3:** Descriptive statistics for model variables.

	**N**	**Frequency or Mean**
**Demographics**		
**Gender (Female)**	793	46%
**Institution (Emory University)**	892	59%
**Underrepresented Minority**	892	13%
**U.S. Citizen**	793	62%
**PhD Student**	892	63%
**Career Interests and Efficacy**		
**Pursuing Non-academic/Non-research Career**	823	72%
**Mean: Career Development Search Process Efficacy**	865	3.47
**Institutional Context**		
**Perceived High Program Support**	829	77%
**Perceived High Advisor Support**	781	78%
**Traditional Career Development Strategy**		
**Sought Career Advice from Advisor**	873	72%
**Sought Career Advice from other Faculty Member**	863	63%
**Other (Non-Traditional) Career Development Strategy**		
**Discussed Career Plans with Family**	879	85%
**Read Career-Related Books, Articles, and/or Online Sources**	873	74%
**Attended a Career-related Event at Own Institution**	867	62%
**Discussed Career Plans with Non-Academic Professionals**	858	56%
**Attended a Career Planning Course not for Credit**	854	27%
**Attended a Career-related Event not at Own Institution**	854	24%
**Participated in the Atlanta BEST Program**	892	23%
**Interned (Not at Institution)**	858	10%
**Job Shadowed**	847	9%
**Interned at Institution**	855	8%
**Attended a Career Planning Course for Credit**	858	6%

Understanding CDSE is central to this research. On average, trainees reported being minimally to moderately confident (3.47 on a five point scale). In the SCCT model, contextual factors are important for career search efficacy. Our results show that the majority of respondents report strong support from their programs (77%) and advisors (78%) for their career goals.

Finally, our descriptive results also show trainees actively engaging in their search for career-relevant resources (Table 1). In terms of the traditional support mechanism for doctoral trainees, almost three-quarters have sought career advice from their advisor, with slightly fewer seeking career advice from another faculty member. On average, respondents reported having participated in about four of the 11 different career development activities listed in Table 1. While it is not certain as to whether these resources are tailored for non-academic careers, academic careers, or both, it does show that some doctoral trainees actively seek career-related resources beyond those of their advisor or other faculty. While the majority of trainees seek support from family and other resources (online resources, books and so on), far fewer have taken advantage of opportunities to intern or job shadow. Notably about half have talked with non-academic professionals about career issues. What is not clear is whether the motivation to seek these alternative resources is driven by career interests, or lack of available resources, or other factors. We address this below.

### Structural equation model for career relevant resources

The descriptive results above suggest some important differences related to resources and career search processes for trainees seeking non-academic careers. To understand the relationship between the factors that matter for CDSE as well as job search strategies, we use a series of structural equation models. This analytical approach reveals three types of relationships between our independent and dependent variables: direct, indirect, and total effects. Direct effects are those effects on the dependent variable that are unmediated by any other independent variable in the model. Indirect effects are effects of an independent variable on the dependent variable mediated by at least one other variable. In our analysis we are particularly interested in how interest in *non-academic careers mediates career development search efficacy*. Total effects are the sum of the direct and indirect effects [46]. We grouped the various career development strategies into two separate categories: traditional sources (typical academic sources) and less-traditional sources of career development.

#### Career self-management: Traditional career development strategies

Traditional sources of career development were defined as those activities where a trainee sought career advice from their advisor/supervisor or another faculty member (expected standard practice in academic settings). The results of our analysis (Tables 3 and 4) show findings consistent with the literature. Theoretically, the SCCT emphasis on efficacy led us to assess the role of career search efficacy as a mediating variable for the career strategies that trainees pursue. Our results show that trainees with non-academic career goals not only have lower career development/search process efficacy, but are also less likely to seek career advice from either their advisor (p<0.01) (Table 4) or from other faculty members (p<0.1) (Table 5). However, varying levels of self-efficacy did not matter in seeking an advisor’s support (but career interests did). However, it was important in whether a trainee sought advice from another faculty member. Lent and Brown [14] argue that this confidence in career development activities affects career goals and actions, which in our case is significant for graduate students and postdocs in the biomedical sciences. Contrary to the factors that matter for seeking out advice from advisors, seeking out advice from other faculty members was driven by the career search efficacy of trainees–those who had a higher career search efficacy were significantly more likely to seek out advice from other academic faculty (p<0.01).

**Table 4:** Structural equation model. Advisor advice career development strategy.

	**Total Effects**	**Direct Effects**	**Indirect Effects**
**Seeking Advisor Advice**			
**Career Search Process Efficacy**	-0.0014	-0.0014	0.0000
**Non-research/Academic Career Path**	-0.1039**	-0.1040**	0.0002
**Institution**	0.0282	0.0281	0.0000
**Perceived Strong Program Support**	0.1169**	0.1173**	-0.0004
**Perceived Strong Advisor Support**	0.2446***	0.2447***	-0.0000
**Female**	-0.0073	-0.0073	0.0000
**Underrepresented Minority**	0.0524	0.0524	-0.0000
**Citizen**	0.1793***	0.1792***	0.0001
**PhD Student**	-0.1626***	-0.1627***	0.0002
**Trainee Career Search Process Efficacy**			
**Non-research/academic Career Path**	-0.1087**	-0.1087**	-
**Institution**	-0.0276	-0.0276	-
**Perceived Strong Program Support**	0.296***	0.296***	-
**Perceived Strong Advisor Support**	0.0129	0.0129	-
**Female**	-0.0092	- 0.0092	-
**Underrepresented Minority**	0.0067	0.0067	-
**Citizen**	-0.0381	-0.0381	-
**PhD Student**	-0.1081**	-0.1081**	-

* p<0.05

**p<0.01

***p<0.001

**Table 5:** Structural equation model. Non-advisor faculty advice career development strategy.

	**Total Effects**	**Direct Effects**	**Indirect Effects**
**Seeking Non-Advisor Faculty Advice**			
**Career Search Process Efficacy**	0.1101**	0.1101*	0.0000
**Non-research/Academic Career Path**	-0.0667	-0.0559	-0.0108
**Institution**	0.0969*	0.1002*	-0.0032
**Perceived Strong Program Support**	0.0618	0.0306	0.0312**
**Perceived Strong Advisor Support**	0.0569	0.0536	0.0033
**Female**	-0.0056	-0.0047	-0.0009
**Underrepresented Minority**	0.0857*	0.0851*	0.0006
**Citizen**	0.2589***	0.2633***	-0.0043
**PhD Student**	-0.1023*	-0.0891*	-0.0132*
**Trainee Career Search Process Efficacy**			
**Non-research/Academic Career Path**	-0.0977**	-0.0977**	-
**Institution**	-0.0288	-0.0288	-
**Perceived Strong Program Support**	0.2832***	0.2832***	-
**Perceived Strong Advisor Support**	0.0302	0.0302	-
**Female**	-0.0088	-0.0088	-
**Underrepresented Minority**	0.0057	0.0057	-
**Citizen**	-0.0389	-0.0389	-
**PhD Student**	-0.1199**	-0.1199**	-

* p<0.05

**p<0.01

***p<0.001

Interestingly, our results also show that a trainee’s perception of high program support for career goals had direct and significant effects on their career development search efficacy, while perceived advisor support was not significant at all. It may be that perceived program support for career goals enables trainees to develop a broader support base within their graduate programs (as opposed to being driven by dissatisfaction with one’s advisor).

Regarding demographics, race/ethnicity was significant in whether the trainee sought advice from a faculty member other than their advisor (underrepresented minority trainees (URM) were more likely to pursue this resource than non-URM trainees). However, this was not the case for trainee CDSE, or the likelihood of seeking career advice from one’s advisor. Notably, there were no gender effects in the likelihood of seeking advice from one’s advisor (coefficient was negative but not significant). Citizenship however had highly significant and positive direct effects on advice seeking from advisors and other faculty members, suggesting that foreign students and postdocs may experience additional barriers in interacting with faculty. Notably, citizenship had no significant effects on CDSE. Overall our results suggest that seeking out advice from an advisor and academic faculty generally occurs when the career goals are supported, likely aligning with expectations of the program and advisor/supervisor.

#### Career self-management: Adaptive career development strategies

Self-adaptation of career development processes is central to the newly adapted Social Cognitive Career Model [14]. In the academic setting, adaptation of career development may involve seeking out resources beyond those of the advisor or other faculty member, both on and off of campus. Our results above prompted a follow-up question–if there are individuals who are less likely to seek support from their advisor or other faculty members (traditional sources of career development), then what strategies, if any, are they pursuing to improve their career development?

In our models, we find significant direct, indirect, and total effects on the factors driving trainees to pursue different types of career development activities. Results are summarized in Table 6, including providing a summary of Tables 4 and 5 for convenience (detailed statistical tables for the remaining models may be found in the supplemental materials for this paper: S1 Appendix of Supporting Tables).

**Table 6:** Overall structural equation results. Total effects.

**Dependent Variable (each for separate model)**^**1**^														
**Career Development/Search Strategies**														
**Predictors**	Trainee Career Search Efficacy	Sought Career Advice from Advisor/ Supervisor	Sought Career Advice from other Faculty Member	Discussed Career Plans w/ Non-Academic Professionals	Discussed Career Plans with Family	Attended a Career-related Event at Own Institution	Attended a Career-related Event not at Your Institution	Read Books, Articles, and/or Online Sources	Participated in Atlanta BEST	Interned at Institution	Interned outside own Institution	Job Shadowed	Attended a Career Planning Course for Credit	Attended a Career Planning Course not for Credit
**Trainee Career Search Efficacy**		**-**	+ **	+ **	**+**	+ *	+ ***	**+**	**+**	**+**	**+**	+ *	+ *	+ *
**Non-research/ non-academic career interest**	- **	- **	**-**	**+**	**+**	**+**	**+**	**+**	+ **	**-**	**+**	**-**	**-**	**-**
**Perceived** **High Program Support**	+ ***	+ **	**+**	**+**	+ *	**+**	**+**	**+**	**-**	**-**	**+**	**-**	**+**	**-**
**Perceived High Advisor Support**		+ ***	**+**	**+**	**-**	**+**	**-**	**-**	**+**	**+**	**+**	**+**	**+**	**-**
**Institution**		**+**	+ *	**+**	**+**	**+**	**+**	**+**	+ ***	+ *	**-**	**+**	**+**	+ **
**PhD Student**	- **	- ***	- *	**-**	- *	**-**	**-**	- *	+ *	**+**	+ *	**-**	**-**	**-**
**Female**		**-**	**-**	**+**	**+**	**+**	**+**	**+**	**+**	**-**	**+**	**-**	**+**	**+**
**Under-represented Minority**		**+**	+ *	**+**	**+**	**-**	**-**	**-**	**+**	**-**	**+**	**+**	**+**	**+**
**U.S. Citizen**		+ ***	+ ***	**+**	+ **	+ *	**-**	**+**	**+**	**+**	**-**	**+**	**-**	**+**

^1^ Detailed statistical models for each column are provided in the appendix as supplemental tables in the order in which they appear here.

+ Positive effect on strategy

- Negative effect on trainee career search strategy

* P < 0.05

** P < 0.01

*** P < 0.001

Of the eleven nontraditional career development strategies that trainees were asked whether they had pursued (activities excluding seeking advisor or other academic faculty advice), only one was significant: having participated in the Atlanta BEST (Broadening Experiences in Scientific Training) program (p<0.01), a highly promoted program targeted to preparation for a broad set of non-academic careers. These results suggest that trainees are searching for alternative sources of career development, as a clear alternative resource (like BEST) for non-academic career preparation may serve as an adaptive mechanism for trainees with those interests.

Notably, trainee career development search efficacy appears to be driving the search for career development resources beyond those of one’s advisor and other faculty. We find that CDSE had strong positive effects on several of the career resources noted, including seeking advice from non-academics (p < .01) as well as taking career development courses (p < .05). These results suggest that developing mechanisms that build the CDSE of trainees with non-academic career interests may have important outcomes, including providing the foundation for seeking and tailoring resources for one’s own career interests.

Consistent with the models that addressed seeking traditional resources, very few gender or race/ethnicity effects were observed in which nontraditional career development strategies individuals pursued. Further, PhD students do seem to pursue different resources than do postdocs, and perceived program or advisor support has no significant effect on whether a trainee pursues any of these varied resources (with the exception of talking with one’s family about career interests). Overall, the results underscore the importance of an individual’s career development search efficacy in seeking career development resources.

## Discussion

It is likely that the percentage of biomedical doctoral students pursuing non-academic careers will continue to increase. Research on what drives these interests and pathways will continue to delve into the experiences, interests, and priorities of early career doctoral recipients in science [4]. We believe that our study complements that stream of research by offering a relevant contribution to the understanding of career transitions in science. As Lent and Brown [14] noted in their motivation to update their Social and Cognitive Career Theory model, the majority of career development studies have focused on *where* individuals ended up in terms of career outcomes. Fewer studies have addressed the “*process”* aspects of career development, and the factors that contribute to this process. It is not enough to know *whether and why* doctoral trainees are interested in careers outside of academia, we must also know *how* they might best achieve their goals. Career search efficacy addresses the capacity and related ability of individuals to take the necessary steps toward achieving their career goals [14]. The purpose of our study was to specifically assess the strategies that doctoral trainees adapt to pursue career interests that fall outside of traditional academic pathways. Do the contexts in which they work and study matter? In these situations, do individuals adapt? Our results suggest that the answer is somewhat mixed. We find that the context in which trainees work and study matters for career search efficacy, and some career development strategies they may pursue, but we also find that there are other factors beyond career interest that additionally drive adaptation.

Our results regarding trainee perceptions of institutional support (program support) show that how trainees perceive their support systems (or lack thereof) has a strong direct effect on their career search efficacy, as well as the types of career development strategies they adopt. These results are consistent with the work of a variety of researchers, including those who focus on the SCCT [1, 6, 14, 15]. Our work is consistent with Lent and colleagues’ work [14, 15] that links institutional support and career efficacy [1] which in turn affects career outcomes [1, 16, 18]. While this may be true, our results provide a preliminary sense of *why* this may be the case. We find the level of career search efficacy to not only mediate the effects of different variables, like institutional support and career interests for seeking advice with faculty other than advisor, but also to be a significant factor for the type of career development strategies individuals pursue. Additionally, consistent with Saks and Ashforth [21], this efficacy is driven largely by the trainees’ perceived level of institutional support along with their career interests.

Another key finding of this research regards the role of career interests and social capital provided by advisors/postdoc supervisors. Current research suggests that if individuals perceive a lack of support for their career goals, then trainees may need alternate mechanisms and resources to achieve their goals [4]. The work that we present further emphasizes the importance of this type of support. Trainees’ perceived institutional support (program support) drove not only the career search efficacy of individuals, but also the strategies they pursued. Trainees with non-academic career goals are not only likely to perceive lower levels of support from their advisors and programs, but they are also less likely to seek advice from their advisor or other faculty. It provides some evidence that trainees with non-academic career interests experience serious barriers to developing resources to help them in their career search and development process. It opens the door to additional research on access and barriers to career-relevant social capital at this career transition stage.

We also find that career search efficacy seems to result in the ability of trainees to adapt and access resources relevant to their interests, such as from not only advisors and other faculty, as well as other external resources and opportunities. Those with higher levels of career search efficacy not only sought advice from faculty but also pursued activities that were tailored to building the career development search efficacy for broader career interests (attending workshops and seeking out advice outside of the academic setting). This aligns with the work of Saks and Ashforth [21] who argue that these individuals are adapting to a lack of control by pursuing different strategies as a means of gaining control over their career outcomes. Though we know that efficacy is a direct adaptive mechanism, where behaviors of individuals help them cope with contextual challenges (Lent & Brown, 2013), there is no need to adapt one’s career development/search process at all if individuals possess adequate efficacy. And, as shown here and consistent with prior studies, this efficacy is largely driven by institutional support [21]. But, notably, we do not find that trainees with non-academic career interests exhibit this adaptive behavior. In fact, it is unclear of whether the resources that trainees with broad career interests access given that they do not seek advice from their advisor or other faculty, and do not access a range of other career development resources. Our analysis pointed to efficacy, not career interests, as a driver for seeking out other resources. Yet, trainees with non-academic career interests have significantly lower career search efficacy.

Our findings have some important implications and demonstrate an obvious gap in the resources and needs of trainees with broader career interests. This gap could be the result of a lack of career development resources designed to address a variety of career types, or that these resources are not well known or utilized across programs and/or students. Existing research points to the culture of academe [47] and complexity of career choice as compounding factors in minimizing this gap, with scholars like Gibbs and Griffin [48] highlighting the need for reforms to be institutional in nature [48]. Our results are salient, as they emphasize the role of the institution in driving career search efficacy and in affecting the career development activities of individuals. Institutional cultures are important in providing contextual support, as described by Lent and Brown [14]. However, given these findings, a lack of advisor support may not be as problematic as assumed given the relatively stronger findings specific to perceived program support as important for trainee career search efficacy. Perhaps, as the work of Layton, Brandt, Freeman, Harrell, Hall, and Sinche [49] suggests, this is because other factors (like career characteristics) are more influential than advisor influence in career choice. A limitation of our work is that the source of this perception of advisor support is not clear (for example, it may reflect a number of things, from staff, to peers, to overall culture and environment). Nevertheless, institutional cultural impediments (such as how program culture affects doctoral student career development) are less understood overall in research and should be explored further.

Given the importance of institutional support in our models, our results suggest a clear role for institutional solutions in addressing the needs and interests of trainees who do not want to pursue an academic or research-intensive career. The U.S. federal government role in supporting the development of a STEM workforce has been evident in considerable investments in addressing the attraction, retention, and advancement of individuals to STEM disciplines and careers. Scientific and related research capacity are supported through programs targeted to institutional change (NSF ADVANCE), doctoral students (dissertation fellowships), post-doctoral fellows (research fellowships and postdoctoral mentoring plan requirements in federal research awards), and early career researchers (Early Career Awards). The newest initiatives that target non-academic careers are an important part of this overall portfolio. Our research, and other studies (e.g. [4]) addressing non-academic career interests point to the need for these types of resources for doctoral trainees. Finally, from a research perspective, researchers focused on the transition of doctoral trainees in the sciences should continue to delve into differences in experiences, resources, and outcomes, and how they vary by career aspirations.

### Limitations

Our study has a couple of limitations worth noting. First, the data for this study rely on perceptions of institutional and advisor support (briefly mentioned above). The origins or drivers of these perceptions is less understood and therefore does not allow for a complete picture of the support an individual might receive. Second, the data were gathered from a survey that asks specifically about career development activities in a single year. The frequency of activities or helpfulness of each type of activity is unknown, thereby potentially affecting rationales for pursuing or not pursuing certain career development opportunities.

### Implications for future research and practice

There are a number of implications from our study that are relevant to future research. First, this research presents opportunities for future work exploring more of the complex dynamics of career search and development efficacy in the sciences. Our research has shown the importance of programmatic support, yet what constitutes this positive culture is not well understood. Further, there is opportunity to address the organization and other factors relevant to driving the development of academic cultures that are supportive for the broad range of career interests. Second, our study is specific to the biomedical field. There is an opportunity to conduct a similar study in other disciplines to see how results hold. This could be particularly enlightening to see how different variables like gender vary in their relationship with CDSE and career interests in different fields. Third, we find minimal demographic effects in terms of career adaptation behavior, while other studies suggest that race and gender can have an effect [50]. In terms of career adaptation, future work should further explore the role that personal characteristics like race and citizenship and gender have on perceptions of support. This may guide on understanding on how support is both perceived and what types trends exist concerning adaptive behavior.

Finally, from a practical perceptive, it will be important to understand which institutional or programmatic aspects are most relevant to trainees in the biomedical sciences (e.g. curriculum and administrative changes to support different types of course work or advising adjustments) in order for universities to provide appropriate resources. Further, how might these resources be most effectively implemented? Are career development resources most effective at the unit/department or university level, or would a cohort/intensive approach be more effective? Changing job markets coupled with changing trainee career interests may push universities to develop support mechanisms for doctoral trainees and their career development processes.

## Supporting information

S1 Appendix Supporting tables. (DOCX)
