# Correction: The "new normal": Adapting doctoral trainee career preparation for broad career paths in science

**DOI:** 10.1371/journal.pone.0181294

**Published:** 2017-07-07

**Authors:** Rebekah St. Clair, Tamara Hutto, Cora MacBeth, Wendy Newstetter, Nael A. McCarty, Julia Melkers

The following information is missing from the Funding section: The Atlanta BEST Program is supported, in part, by the Laney Graduate School at Emory University.
