# Supplementary material for: The “new normal”: Adapting doctoral trainee career preparation for broad career paths in science
Source: PLoS One. 2017 May 24;12(5):e0177035. doi: 10.1371/journal.pone.0177035 (PMC5443479; doi:10.1371/journal.pone.0177035)
Supplement: S1 Appendix — (DOCX) [file pone.0177035.s001.docx]

**S1 Appendix. SUPPLEMENTAL MATERIALS**

| **S1 Table: Structural Equation Model**  **Process Efficacy and Non-Academic Professional Advice Career Development Strategy** | | | |
| --- | --- | --- | --- |
| **Seeking Non-Academic Professional Advice** | | | |
|  | **Total Effects** | **Direct Effects** | **Indirect Effects** |
| Career Development Search Process Efficacy | 0.1217** | 0.1217** | 0.0000 |
| Non-research/Academic Career Path | 0.0447 | 0.0569 | -0.0122* |
| Institution | 0.0010 | -0.0057 | -0.0047 |
| Perceived Strong Program Support | 0.0039 | -0.0308 | 0.0309** |
| Perceived Strong Advisor Support | 0.0095 | -0.0057 | 0.0036 |
| Female | 0.0163 | 0.0161 | 0.0001 |
| Underrepresented Minority | 0.0544 | 0.0542 | 0.0002 |
| Citizen | 0.0520 | 0.0562 | -0.0042 |
| PhD Student | -0.0366 | -0.0222 | -0.0144* |
| **Possessing Process Efficacy** | | | |
| Non-research/Academic Career Path | -0.0999** | -0.0999** | - |
| Institution | -0.0389 | -0.0389 | - |
| Perceived Strong Program Support | 0.2847*** | 0.2847*** | - |
| Perceived Strong Advisor Support | 0.0308 | 0.0308 | - |
| Female | 0.0009 | 0.0009 | - |
| Underrepresented Minority | 0.0014 | 0.0014 | - |
| Citizen | -0.0343 | -0.0343 | - |
| PhD Student | -0.1184** | -0.1184** | - |
| * p<.05; **p<.01; ***p<.001 |  |  |  |

| **S2 Table: Structural Equation Model**  **Process Efficacy and Family Advice Career Development Strategy** | | | |
| --- | --- | --- | --- |
| **Seeking Family Advice** | | | |
|  | **Total Effects** | **Direct Effects** | **Indirect Effects** |
| Career Development Search Process Efficacy | 0.0163 | 0.0163 | 0.0000 |
| Non-research/Academic Career Path | 0.0551 | 0.0569 | -0.0018 |
| Institution | 0.0439 | 0.0443 | -0.0003 |
| Perceived Strong Program Support | 0.0905* | 0.0859 | 0.0046 |
| Perceived Strong Advisor Support | -0.0097 | -0.0102 | 0.0005 |
| Female | 0.0280 | 0.0281 | -0.0001 |
| Underrepresented Minority | 0.0232 | 0.0231 | 0.0001 |
| Citizen | 0.1146** | 0.1151** | -0.0005 |
| PhD Student | -0.0914* | -0.0898* | -0.0016 |
| **Possessing Process Efficacy** | | | |
| Non-research/Academic Career Path | -0.1089** | -0.1089** | - |
| Institution | -0.0168 | -0.0140 | - |
| Perceived Strong Program Support | 0.2816*** | 0.2813*** | - |
| Perceived Strong Advisor Support | 0.0305 | 0.0336 | - |
| Female | -0.0044 | -0.0044 | - |
| Underrepresented Minority | 0.0046 | 0.0046 | - |
| Citizen | -0.0312 | -0.0312 | - |
| PhD Student | -0.0991* | -0.0991*** | - |
| * p<.05; **p<.01; ***p<.001 |  |  |  |

| **S3 Table: Structural Equation Model**  **Process Efficacy and Attending Career Events at Institution Career Development Strategy** | | | |
| --- | --- | --- | --- |
| **Attending Career Events at Institution** | | | |
|  | **Total Effects** | **Direct Effects** | **Indirect Effects** |
| Career Development Search Process Efficacy | 0.0828* | 0.0828* | 0.0000 |
| Non-research/Academic Career Path | 0.0118 | 0.0209 | -0.0091 |
| Institution | 0.0361 | 0.0383 | -0.0022 |
| Perceived Strong Program Support | 0.0226 | -0.0016 | 0.0243* |
| Perceived Strong Advisor Support | 0.0189 | 0.0173 | 0.0017 |
| Female | 0.0559 | 0.0566 | 0.0007 |
| Underrepresented Minority | -0.0249 | -0.0249 | 0.0000 |
| Citizen | 0.1044* | 0.1070* | -0.0026 |
| PhD Student | -0.0760 | -0.0668 | -0.0092 |
| **Possessing Process Efficacy** | | | |
| Non-research/Academic Career Path | -0.1099** | -0.1099** | - |
| Institution | -0.0271 | -0.0271 | - |
| Perceived Strong Program Support | 0.2929*** | 0.2929*** | - |
| Perceived Strong Advisor Support | 0.0203 | 0.0203 | - |
| Female | -0.0079 | -0.0079 | - |
| Underrepresented Minority | 0.0003 | 0.0003 | - |
| Citizen | -0.0319 | 0.0319 | - |
| PhD Student | -0.1112** | -0.1112** | - |
| * p<.05; **p<.01; ***p<.001 |  |  |  |

| **S4 Table: Structural Equation Model**  **Process Efficacy and Attending Career Events Not at Institution Career Development Strategy** | | | |
| --- | --- | --- | --- |
| **Attending Career Events Not at Institution** | | | |
|  | **Total Effects** | **Direct Effects** | **Indirect Effects** |
| Career Development Search Process Efficacy | 0.1590*** | 0.1590*** | 0.0000 |
| Non-research/Academic Career Path | 0.0198 | 0.0362 | -0.0164* |
| Institution | 0.0003 | 0.0046 | -0.0042 |
| Perceived Strong Program Support | 0.0606 | 0.0137 | 0.0469** |
| Perceived Strong Advisor Support | -0.0629 | -0.0666 | 0.0037 |
| Female | 0.0355 | 0.0368 | -0.0014 |
| Underrepresented Minority | -0.0678 | -0.0695 | 0.0017 |
| Citizen | -0.0075 | -0.0024 | -0.0051 |
| PhD Student | -0.0312 | -0.0132 | -0.0179* |
| **Possessing Process Efficacy** | | | |
| Non-research/Academic Career Path | -0.1029** | -0.1029** | - |
| Institution | -0.0266 | -0.0266 | - |
| Perceived Strong Program Support | 0.2946*** | 0.2946*** | - |
| Perceived Strong Advisor Support | 0.0231 | 0.0231 | - |
| Female | -0.0086 | -0.0086 | - |
| Underrepresented Minority | 0.0108 | 0.0108 | - |
| Citizen | -0.0322 | -0.0322 | - |
| PhD Student | -0.1127** | -0.1127** | - |
| * p<.05; **p<.01; ***p<.001 |  |  |  |

| **S5 Table: Structural Equation Model**  **Process Efficacy and Personal Reading/Research Career Development Strategy** | | | |
| --- | --- | --- | --- |
| **Personal Reading/Research** | | | |
|  | **Total Effects** | **Direct Effects** | **Indirect Effects** |
| Career Development Search Process Efficacy | 0.0405 | 0.0405 | 0.0000 |
| Non-research/Academic Career Path | 0.0139 | 0.0183 | -0.0038 |
| Institution | 0.0359 | 0.0373 | -0.0011 |
| Perceived Strong Program Support | 0.0647 | 0.0535 | 0.0113 |
| Perceived Strong Advisor Support | -0.0705 | -0.0719 | 0.0014 |
| Female | 0.0349 | 0.0354 | -0.0005 |
| Underrepresented Minority | -0.0085 | -0.0086 | 0.0000 |
| Citizen | 0.0663 | 0.0676 | -0.0014 |
| PhD Student | -0.0898* | -0.0851 | -0.0047 |
| **Possessing Process Efficacy** | | | |
| Non-research/Academic Career Path | -0.1072** | -0.1072** | - |
| Institution | -0.0324 | -0.0324 | - |
| Perceived Strong Program Support | 0.2783*** | 0.2783*** | - |
| Perceived Strong Advisor Support | 0.0352 | 0.0352 | - |
| Female | -0.0122 | -0.0122 | - |
| Underrepresented Minority | 0.0007 | 0.0007 | - |
| Citizen | -0.0339 | -0.0339 | - |
| PhD Student | -0.1163** | -0.1163** | - |
| * p<.05; **p<.01; ***p<.001 |  |  |  |

| **S6 Table: Structural Equation Model**  **Process Efficacy and Participating in BEST Career Development Strategy** | | | |
| --- | --- | --- | --- |
| **Participating in BEST** | | | |
|  | **Total Effects** | **Direct Effects** | **Indirect Effects** |
| Career Development Search Process Efficacy | 0.0388 | 0.0388 | 0.0000 |
| Non-research/Academic Career Path | 0.1049** | 0.1092** | -0.0042 |
| Institution | 0.2709*** | 0.2715*** | -0.0007 |
| Perceived Strong Program Support | -0.0437 | -0.0548 | 0.0111 |
| Perceived Strong Advisor Support | 0.0313 | 0.0303 | 0.0009 |
| Female | 0.0299 | 0.0307 | -0.0003 |
| Underrepresented Minority | 0.0452 | 0.0449 | 0.0002 |
| Citizen | 0.0664 | 0.0673 | -0.0009 |
| PhD Student | 0.0935* | 0.0974* | -0.0039 |
| **Possessing Process Efficacy** | | | |
| Non-research/Academic Career Path | -0.1094** | -0.1094** | - |
| Institution | -0.0177 | -0.0177 | - |
| Perceived Strong Program Support | 0.2856*** | 0.2856*** | - |
| Perceived Strong Advisor Support | 0.0252 | 0.0252 | - |
| Female | -0.0082 | -0.0082 | - |
| Underrepresented Minority | 0.0055 | -0.0055 | - |
| Citizen | -0.0253 | -0.0253 | - |
| PhD Student | -0.1006* | -0.1006* | - |
| * p<.05; **p<.01; ***p<.001 |  |  |  |

| **S7 Table: Structural Equation Model**  **Process Efficacy and Interning at Institution Career Development Strategy** | | | |
| --- | --- | --- | --- |
| **Interning at Institution** | | | |
|  | **Total Effects** | **Direct Effects** | **Indirect Effects** |
| Career Development Search Process Efficacy | 0.0485 | 0.0485 | 0.0000 |
| Non-research/Academic Career Path | -0.0664 | -0.0617 | -0.0047 |
| Institution | 0.1070* | 0.1085* | -0.0015 |
| Perceived Strong Program Support | -0.0175 | -0.0314 | 0.0139 |
| Perceived Strong Advisor Support | 0.0564 | 0.0549 | 0.0014 |
| Female | -0.0080 | -0.0076 | 0.0004 |
| Underrepresented Minority | -0.0499 | -0.0503 | 0.0004 |
| Citizen | 0.0457 | 0.0474 | -0.0016 |
| PhD Student | 0.0073 | 0.0126 | -0.0053 |
| **Possessing Process Efficacy** | | | |
| Non-research/Academic Career Path | -0.0970** | -0.0970** | - |
| Institution | -0.0306 | -0.0306 | - |
| Perceived Strong Program Support | 0.2873*** | 0.2873*** | - |
| Perceived Strong Advisor Support | 0.0289 | 0.0289 | - |
| Female | -0.0081 | -0.0081 | - |
| Underrepresented Minority | 0.0089 | 0.0089 | - |
| Citizen | -0.0336 | -0.0336 | - |
| PhD Student | -0.1098** | -0.1034** | - |
| * p<.05; **p<.01; ***p<.001 |  |  |  |

| **S8 Table: Structural Equation Model**  **Process Efficacy and Interning outside of Institution Career Development Strategy** | | | |
| --- | --- | --- | --- |
| **Interning outside of Institution** | | | |
|  | **Total Effects** | **Direct Effects** | **Indirect Effects** |
| Career Development Search Process Efficacy | 0.0619 | 0.0619 | 0.0000 |
| Non-research/Academic Career Path | 0.0663 | 0.0723 | -0.0060 |
| Institution | -0.0736 | -0.0717 | -0.0018 |
| Perceived Strong Program Support | 0.0272 | 0.0095 | 0.0176 |
| Perceived Strong Advisor Support | 0.0414 | 0.0394 | 0.0019 |
| Female | 0.0126 | 0.0130 | -0.0004 |
| Underrepresented Minority | 0.0151 | -0.0145 | 0.0007 |
| Citizen | -0.0082 | -0.0060 | -0.0022 |
| PhD Student | 0.0900* | 0.0967* | -0.0066 |
| **Possessing Process Efficacy** | | | |
| Non-research/Academic Career Path | -0.0973** | -0.0973** | - |
| Institution | -0.0295 | -0.0295 | - |
| Perceived Strong Program Support | 0.2851*** | 0.2851*** | - |
| Perceived Strong Advisor Support | 0.0321 | 0.0321 | - |
| Female | -0.0068 | -0.0068 | - |
| Underrepresented Minority | 0.0109 | 0.0109 | - |
| Citizen | -0.0359 | -0.0359 | - |
| PhD Student | -0.1074* | -0.1074* | - |
| * p<.05; **p<.01; ***p<.001 |  |  |  |

| **S9 Table: Structural Equation Model**  **Process Efficacy and Job Shadowing Career Development Strategy** | | | |
| --- | --- | --- | --- |
| **Job Shadowing** | | | |
|  | **Total Effects** | **Direct Effects** | **Indirect Effects** |
| Career Development Search Process Efficacy | 0.0986* | 0.0986* | 0.0000 |
| Non-research/Academic Career Path | -0.0067 | 0.0031 | -0.0097 |
| Institution | 0.0608 | 0.0626 | -0.0018 |
| Perceived Strong Program Support | -0.0503 | -0.0784 | 0.0280* |
| Perceived Strong Advisor Support | 0.0321 | 0.0294 | 0.0026 |
| Female | -0.0019 | -.0015 | -0.0005 |
| Underrepresented Minority | 0.0717 | 0.0706 | 0.0011 |
| Citizen | 0.0542 | 0.0565 | -0.0023 |
| PhD Student | -0.0128 | -0.0019 | -0.0109 |
| **Possessing Process Efficacy** | | | |
| Non-research/Academic Career Path | -0.0988** | -0.0988** | - |
| Institution | -0.0181 | -0.0181 | - |
| Perceived Strong Program Support | 0.2840*** | 0.2840*** | - |
| Perceived Strong Advisor Support | 0.0268 | 0.0268 | - |
| Female | 0.0046 | 0.0046 | - |
| Underrepresented Minority | 0.0112 | 0.0112 | - |
| Citizen | -0.0232 | -0.0232 | - |
| PhD Student | -0.1108** | -0.1108** | - |
| * p<.05; **p<.01; ***p<.001 |  |  |  |

| **S10 Table: Structural Equation Model**  **Process Efficacy and Participating in a Course for Credit Career Development Strategy** | | | |
| --- | --- | --- | --- |
| **Participating in a Course for Credit** | | | |
|  | **Total Effects** | **Direct Effects** | **Indirect Effects** |
| Career Development Search Process Efficacy | 0.0985* | 0.0985* | 0.0000 |
| Non-research/Academic Career Path | -0.0413 | -0.0314 | -0.0099 |
| Institution | 0.0166 | 0.0195 | -0.0029 |
| Perceived Strong Program Support | 0.0589 | 0.0305 | 0.0285* |
| Perceived Strong Advisor Support | 0.0338 | 0.0308 | 0.0029 |
| Female | 0.0038 | 0.0042 | -0.0004 |
| Underrepresented Minority | 0.0714 | 0.0707* | 0.0007 |
| Citizen | -0.0385 | -0.0347 | -0.0038 |
| PhD Student | -0.0032 | 0.0079 | -0.0112 |
| **Possessing Process Efficacy** | | | |
| Non-research/Academic Career Path | -0.1000** | -0.1000** | - |
| Institution | -0.0295 | -0.0295 | - |
| Perceived Strong Program Support | 0.2891*** | 0.2891*** | - |
| Perceived Strong Advisor Support | 0.0298 | 0.0298 | - |
| Female | -0.0044 | -0.0044 | - |
| Underrepresented Minority | 0.0074 | 0.0074 | - |
| Citizen | -0.0387 | -0.0387 | - |
| PhD Student | -0.1133** | -0.1133** | - |
| * p<.05; **p<.01; ***p<.001 |  |  |  |

| **S11 Table: Structural Equation Model**  **Process Efficacy and Participating in a Course Not for Credit Career Development Strategy** | | | |
| --- | --- | --- | --- |
| **Participating in a Course Not for Credit** | | | |
|  | **Total Effects** | **Direct Effects** | **Indirect Effects** |
| Career Development Search Process Efficacy | 0.0996* | 0.0996* | 0.0000 |
| Non-research/Academic Career Path | -0.0743 | -0.0636 | -0.0107 |
| Institution | 0.1277** | 0.1306** | -0.0029 |
| Perceived Strong Program Support | -0.0174 | -0.0470 | 0.0296* |
| Perceived Strong Advisor Support | -0.0551 | -0.0577 | 0.0025 |
| Female | 0.0685 | 0.0691* | 0.0005 |
| Underrepresented Minority | 0.0269 | 0.0263 | 0.0006 |
| Citizen | 0.0215 | 0.0254 | -0.0039 |
| PhD Student | -0.0119 | -0.0006 | -0.0113 |
| **Possessing Process Efficacy** | | | |
| Non-research/Academic Career Path | -0.1070** | -0.1070** | - |
| Institution | -0.0291 | -0.0291 | - |
| Perceived Strong Program Support | 0.2970*** | 0.2970*** | - |
| Perceived Strong Advisor Support | 0.0253 | 0.0253 | - |
| Female | -0.0052 | -0.0052 | - |
| Underrepresented Minority | 0.0064 | 0.0064 | - |
| Citizen | -0.0396 | -0.0396 | - |
| PhD Student | -0.1132** | -0.1132** | - |
| * p<.05; **p<.01; ***p<.001 |  |  |  |
